# 一线化疗方案治疗广泛期小细胞肺癌的网状*meta*分析

**DOI:** 10.3779/j.issn.1009-3419.2016.04.02

**Published:** 2016-04-20

**Authors:** 玉洁 陈, 凌霄 陈, 殿胜 钟, 竞 王, 李娜 彭, 鑫 冯

**Affiliations:** 1 300052 天津，天津医科大学总医院肿瘤科 Department of Oncology, Tianjin General Hospital, Tianjin 300052, China; 2 300052 天津，天津医科大学总医院骨科 Department of Orthopedics, Tianjin General Hospital, Tianjin 300052, China; 3 300052 天津，天津医科大学总医院，天津市肺癌研究所 Tianjin Lung Cancer Institute, Tianjin General Hospital, Tianjin 300052, China

**Keywords:** *Meta*分析, 小细胞肺癌, 一线化疗, *Meta*-analysis, Small cell lung cancer, First-line chemotherapy

## Abstract

**背景与目的:**

顺铂/卡波联合伊立替康/依托泊苷双药化疗方案被推荐为治疗广泛期小细胞肺癌（small cell lung cancer of extensive disease, ED-SCLC）的一线化疗方案。我们在网状*meta*分析中，通过合成直接证据和间接证据，对推荐的化疗方案的短期疗效进行排序。

**方法:**

我们在EMBASE、PubMed、CENTRAL及clinicaltrial.gov数据库检出了相关疗效比较的随机对照试验。ROB工具被用于评估纳入研究的质量，Stata 13.1被用于统计学合成。

**结果:**

该研究共纳入10项随机对照研究，共计2, 378名患者。与依托泊苷联合卡铂相比，肿瘤的完全缓解率（complete remission rate, CR）在伊立替康联合卡铂组明显提高（OR=3.33, 95%CI: 1.47-7.54, *P* < 0.05）。伊立替康联合卡铂治疗后CR明显优于依托泊苷联合顺铂（OR=4.09, 95%CI: 1.18-14.12, *P* < 0.05）。

**结论:**

本研究结果显示：伊立替康联合卡铂一线治疗ED-SCLC的疗效优于依托泊苷。

## Abstract (en)

**Background and objective:**

Regimens that combine irinotecan or etoposide with cisplatin or carboplatin have been recommended as first-line regimen for extensive-disease small cell lung cancer (ED-SCLC). In our network *meta*-analysis, we synthesized the direct and indirect lines of evidence to rank the short-term efficacies of these recommended chemotherapy regimens.

**Methods:**

We searched databases, including EMBASE, PubMed, CENTRAL and clinicaltrial.gov, for relevant randomized controlled trials (RCTs) that compared the efficacies of these treatments. A risk of bias tool was used to evaluate the quality of the included studies, whereas Stata 13.1 was used for statistical synthesis.

**Results:**

Our study included 10 RCTs that involved 2, 378 patients. Compared with that of the regimen that combined etoposide and carboplatin, the complete remission rate was significantly higher in the group treated with irinotecan combined with carboplatin. The efficacy of the regimen that combined irinotecan with carboplatin was significantly superior over that of the combination of etoposide and cisplatin.

**Conclusion:**

Our data presented here suggest that the effect of Irinotecan combination with Carboplatin is remarkably superior.

小细胞肺癌（small cell lung cancer, SCLC）的发病率占肺癌的15%，死亡人数占所有肺癌的25%^[1]^。SCLC易出现血行转移，广泛期患者约占SCLC总数的2/3，中位生存时间约为6周，5年生存率低于1%^[2]^。但SCLC对早期化疗及放疗非常敏感，研究^[3, 4]^证明，化疗可以缓解大多数广泛期患者的症状并延长生存时间。过去的二十年中，顺铂/卡铂联合依托泊苷的化疗方案一直被作为治疗广泛期SCLC的标准疗法^[5]^。两项Ⅲ期临床试验（JCOG 9511与SWOG 0124）先后于2002年及2009年比较了依托泊苷与伊立替康分别联合顺铂的化疗方案的有效性、肿瘤应答、总生存期（overall survive, OS）及无疾病进展生存期（progression-free survive, PFS），但两研究结论之间存在较大的争议^[6, 7]^。

根据以前的多项随机对照试验，2015美国国立综合癌症网络（National Comprehensive Cancer Network, NCCN）指南推荐顺铂/卡铂联合依托泊苷/伊立替康作为广泛期小细胞肺癌（small cell lung cancer of extensive disease, ED-SCLC）的一线化疗药物^[8]^。但是，由于目前缺乏几种方案之间的相互比较，最佳的干预措施存在争议。传统的*meta*分析仅能对2个方案的疗效进行比较，而不能根据多个方案的疗效大小对其有效性进行准确的比较并排序^[9]^。Bakalos等^[10]^在2012年运用网状*meta*分析对多种化疗方案治疗SCLC的有效性与患者的耐受性进行了评估。然而，该研究存在一些不足：①没有根据所有方案的疗效大小将反映短期有效性的结局指标进行排序；②对于我们所关注的一线方案，该研究纳入文献较少，使其效能不足且结论不够稳健；③该研究仅对部分反映肿瘤应答情况的结局指标进行了评估，使得其对于短期疗效的评价不够全面。

因此，我们的目的是通过网状*meta*分析对多个化疗方案在治疗ED-SCLC过程中的短期疗效进行排序。通过整合直接证据（头对头试验）和间接证据（通过中间共同变量对非头对头实验进行比较），我们的研究将对各种一线化疗方案的肿瘤应答效果进行排序，得出短期疗效最佳的治疗方案并指导临床实践。

## 1 资料与方法

### 1.1 纳入及排除标准

#### 1.1.1 研究设计

我们的研究仅纳入相关的随机对照试验。对于具有交叉设计的随机对照试验，我们只对洗脱期前的结果进行研究。

#### 1.1.2 研究对象

该研究所纳入的临床试验均以未经治疗的广泛期SCLC患者为研究对象。若随机试验中同时包含局限期和广泛期患者，我们仅提取广泛期患者的相关信息和数据。如果研究中的患者在接受一线化疗方案前接受过手术、放疗或化疗，那么我们排除这类研究。

#### 1.1.3 干预措施

符合纳入标准的随机对照试验需对一线方案（顺铂/卡铂联合依托泊苷/伊立替康）中的至少两种进行比较。对于药物种类相同，但用药剂量、用药时间或给药途径不同的方案，为了简化计算我们均视为同种方案。最后我们会通过敏感性分析和亚组分析对所纳入研究对象的异质性及混杂因素进行校正。

#### 1.1.4 结局指标

该研究以短期疗效作为评价化疗有效性的指标，根据实体肿瘤的疗效评价标准（Response Evaluation Criteria in Solid Tumors, RECIST）^[11]^将其分为：完全缓解（complete remission, CR）、部分缓解（partial remission, PR）、稳定（stable disease, SD）、进展（progressive disease, PD）；无法评估则定为（not evaluable, NE），并记录相应的百分率。此外，考虑到总应答率（overall response rate, ORR）被作为评价临床化疗短期疗效的重要指标，我们将其作为结局指标。

### 1.2 检索策略

我们将“small cell lung cancer”和“randomized controlled trials”及其同义词作为关键词，在EMBASE、PubMed、Cochrane Central Register of Controlled Trials（CENTRAL）以及clinicaltrials.gov四个电子数据库中对文献的标题与摘要进行检索。例如，我们在检索PubMed时应用了如下检索策略：①：small cell lung cancer [mh]；②：oat cell [tiab]；③：SCLC [tiab]；④：① or ② or ③；⑤：（randomized controlled trial [pt] OR controlled clinical trial [pt] OR randomized [tiab] OR placebo [tiab] OR clinical trials as topic [mesh: noexp] OR randomly [tiab] OR trial [ti]）NOT（animals [mh] NOT humans [mh]）；⑥：④ and ⑤。此外我们筛查初筛文献、相关的指南以及综述的参考文献，并将符合条件的文献补充纳入。

### 1.3 文献筛选及信息提取

两位研究员独立的对检出文献的题目、摘要和全文进行核对，意见分歧时交由第三位资深研究员决定。此外我们对文献的排除原因进行了记录。标准化的表格被用于记录纳入研究的关键信息和细节（随机序列的产生、分配隐藏、随访时间等），受试者（肿瘤分期、诊断标准、患者基线特征等），干预措施及对照，结局指标（ORR、CR、PR等），失访情况，利益冲突。同时，我们就研究中不清楚的信息联系了文章的第一或通讯作者。

### 1.4 质量评估

两名研究员通过Cochrane偏倚风险评估工具分别对纳入研究的质量进行了评估^[12]^。每个研究的评估都基于以下7个方面分别进行评估，并按“高”“无法评价”“低”对每项偏倚风险进行评价：①随机序列的产生；②是否采用分配隐藏；③受试者及实施人员是否采用盲法；④结果评价是否采用盲法；⑤不完全的结局指标；⑥选择性结果报告；⑦其他（商业赞助、基线不平衡及早期失访）。

### 1.5 统计分析

考虑到研究间存在的异质性，我们所有的数据分析均采用随机模型来进行。对于有直接比较的研究，我们首先通过STATA V.13.1中的metan命令进行传统的*meta*分析。然后我们通过STATA V.13.1中的mvmeta命令进行网状*meta*分析^[13]^。对于研究间的异质性，我们用*I*^2^来进行测量^[12]^。当*I*^2^≥50%时，我们认为其存在明显的异质性^[12]^。考虑到分析的复杂性，我们在分析中忽略了剂量的不同。当同一研究中有多个剂量组共同存在时，我们选择纳入指南推荐的剂量。对于可能影响研究的变量，例如年龄组成的不同和性别比例的不同，我们通过STATA V.13.1中的mvmeta命令进行*meta*回归来衡量其作用^[14]^。对于不一致性的测量，由于纳入的干预措施无法成环，因此我们不能应用基于环的检验措施和基于全局的检测，只能通过研究内的*I*^2^做一判断^[15]^。所有结局指标均为二分类指标，所以采用比值比（odds ratio, OR）及其95%置信区间（credible interval, CI）来表示。此外我们还进行了每一个指标的预测区间（predictive interval, PrI）的计算。预测区间是一个比置信区间更加保守的指标，可以用于说明是否需要进一步的研究^[15]^。对于最后的结果，我们使用曲线下累积排序概率（surface under the cumulative ranking, SUCRA）来进行计算^[16]^。对于风险偏倚评估为高风险的研究，我们将其剔除进行敏感性分析来探究结果是否稳健。

### 1.6 发表偏倚评价

因为每一个比较纳入的研究数目少于10个，因此我们不进行漏斗图的绘制。

## 2 结果

### 2.1 文献检索及纳入研究情况

优先报告的系统综述和荟萃分析的项目（Preferred Reporting Items for Systematic Reviews and *Meta*-Analyses, PRISMA）流程图（图 1）展示了从文献检索到纳入研究的具体过程。检索日期截止至2015年12月，我们对满足了纳入标准的10篇文献^[6, 7, 17-24]^进行了数据合成和*meta*分析，并对77篇因干预措施不满足纳入要求的文献进行了排除。10项纳入研究的基线特征详见（表 1）。

**1:**
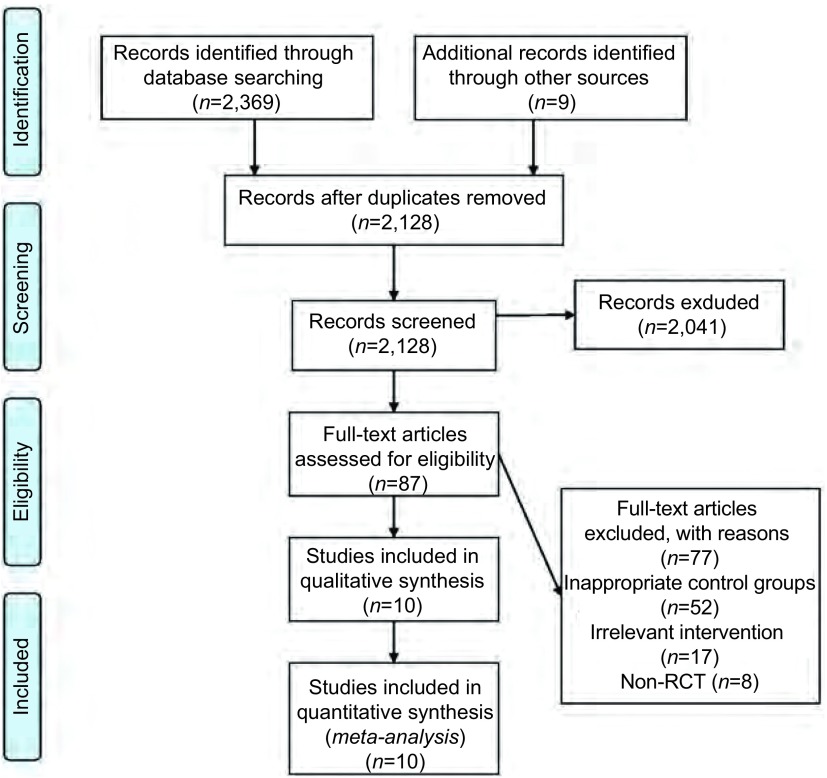
PRISMA流程图 PRISMA flow diagram. PRISMA: Preferred Repor ting Items for Systematic Reviews and *Meta*-Analyses. RCT: Randomized Controlled Trial.

**1:** 纳入研究的基本特征 The characteristics of included studies

Study	Comparators	Country	No. of Patients	Gender (M/F)	Age (yr, median)	Follow-up
Herms 2008^[19]^	iri+car *vs* eto+car	Norway, Sweden	209	138/71	67 *vs* 68	40 weeks
Lara 2009^[6]^	iri+cis *vs* eto+cis	United States	651	370/281	62 *vs* 63	/
Hanna 2006^[23]^	iri+cis *vs* eto+cis	United States	331	189/141	63 *vs* 62	18 month
Noda 2002^[7]^	iri+cis *vs* eto+cis	Japan	154	136/22	63 *vs* 63	1.5 years
Okamoto 2007^[20]^	eto+car *vs* eto+cis	Japan	220	193/27	74 *vs* 73.5	/
Pan 2006^[22]^	iri+cis *vs* eto+cis	China	61	47/14	54 *vs* 51	/
Schmittel 2011^[17]^	iri+car *vs* eto+car	Germany	216	141/75	60 *vs* 63	/
Skarlos 1994^[24]^	eto+cis *vs* eto+car	Greece	61	/	/	2 years
Zatloukal 2010^[18]^	iri+cis *vs* eto+cis	Europe	405	309/96	60 *vs* 61	31.6 months
Schmittel 2006^[21]^	iri+car *vs* eto+car	Germany	70	50/20	59 *vs* 63	21 months
iri: irinotecan; car: carboplatin; eto: etoposide; cis: cisplatin; M: Male; F: Female.						

### 2.2 偏倚风险评估

在纳入的10项研究中，8项研究详细的描述了随机序列的产生，3项研究提及了分配隐藏，所有的研究均对受试者和实施人员实施了盲法，仅有2项研究提及了对结果评价者实施了盲法，所有的研究在不完全结局指标和选择性结局报告上均为低风险，3项研究接受了商业赞助（图 2）。

**2:**
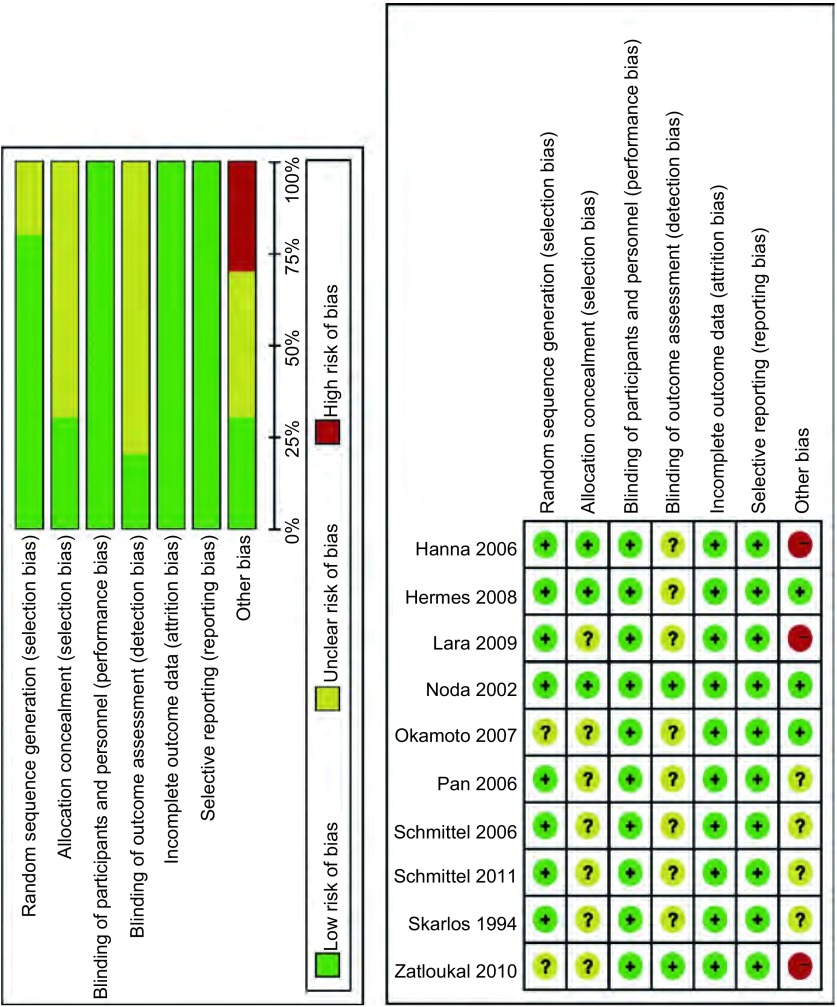
纳入研究的质量评价 Risk of bias for included studies

### 2.3 各一线方案短期疗效*meta*分析结果

#### 2.3.1 ORR

我们对9项随机对照试验中的2, 169例患者及4种指南推荐的一线化疗方案的ORR进行了网状*meta*分析（图 3）。其中，伊立替康联合顺铂：共5项随机对照试验，854例患者；伊立替康联合卡铂：共3项随机对照试验，246例患者；依托泊苷联合顺铂：共7项随机对照试验，888例患者；依托泊苷联合卡铂：共5项随机对照试验，390例患者。结果表明，各方案ORR之间没有明显的统计学差异（表 2）。通过计算比95%CI更为保守的预测区间（95%PrI）（图 4），四种方案结果仍无统计学差异。经异质性检验，各方案之间未见明显的异质性。根据SUCRA曲线进行排序（图 5），四种方案中肿瘤ORR最高的为伊立替康联合卡铂。敏感性分析及*meta*回归均没有改变结果。

**3:**
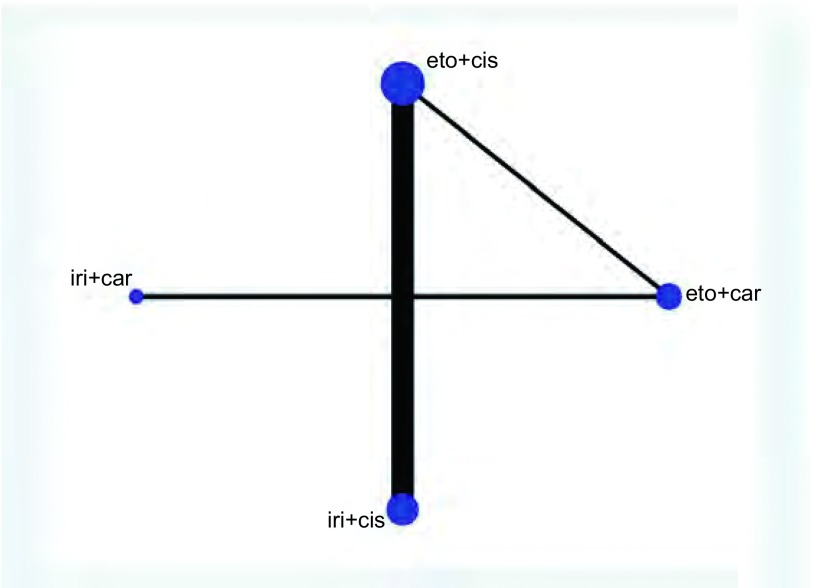
ORR的网状图 Network plot of ORR. ORR: Overall Response Rate.

**2:** ORR的网状*meta*分析结果 The network results of ORR

iri+cis			
0.91 (0.37, 2.20)	iri+car		
1.13 (0.83, 1.56)	1.25 (0.54, 2.89)	eto+cis	
1.03 (0.52, 2.03)	1.13 (0.64, 2.01)	0.91 (0.49, 1.67)	eto+car

**4:**
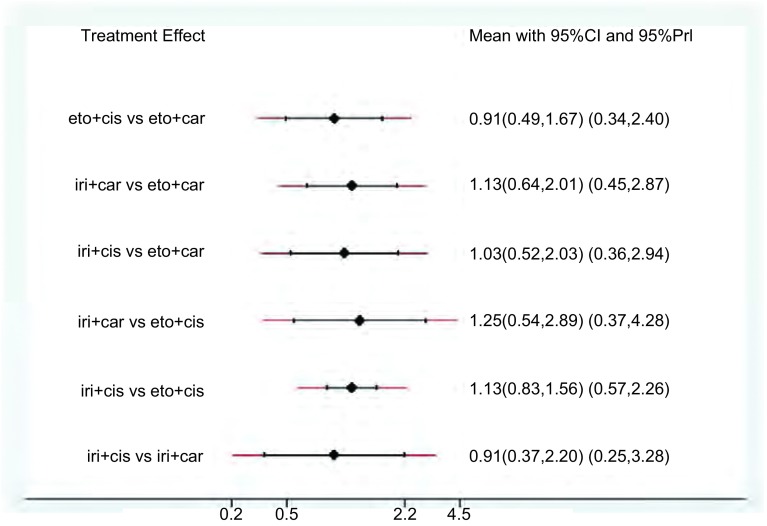
ORR的预测区间图 Predictive interval plot of ORR

**5:**
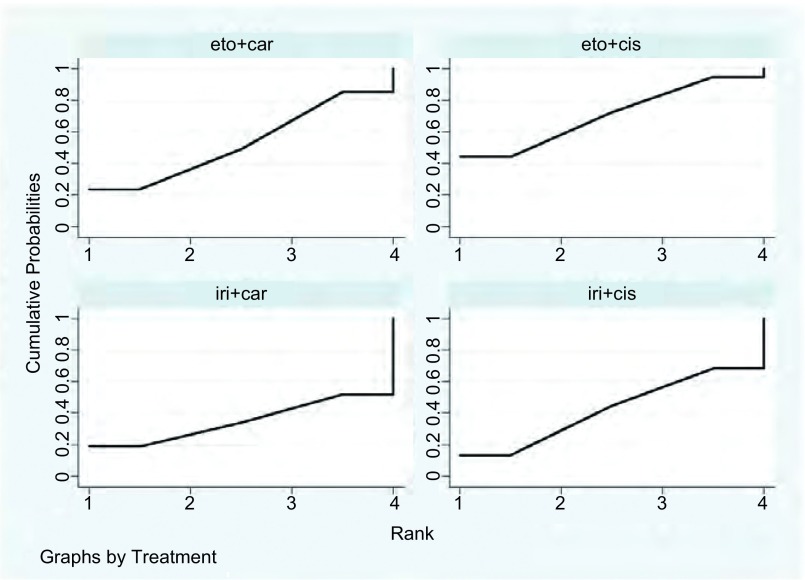
ORR的SUCRA图 SUCRA plot of ORR

#### 2.3.2 CR

8项随机对照试验对接受4种指南推荐的一线化疗方案的1, 396例患者的CR进行了网状*meta*分析。其中，伊立替康联合顺铂：共3项随机对照试验，309名患者；伊立替康联合卡铂:共3项随机对照试验，246例患者；依托泊苷联合顺铂:共5项随机对照试验，451例患者；依托泊苷联合卡铂：共5项随机对照试验，390例患者。经比较，伊立替康联合卡铂较依托泊苷联合卡铂（OR=3.33, 95%CI: 1.47-7.54, *P* < 0.05）及依托泊苷联合顺铂（OR=4.09, 95%CI: 1.18-14.12, *P* < 0.05）有更高的CR，有明显的统计学差异。其余各方案CR之间没有明显的统计学差异。通过计算预测区间（95%PrI），伊立替康联合卡铂的CR仍高于依托泊苷联合卡铂，其余各方案结果比较，无明显统计学差异。异质性检验未见明显的差异。根据SUCRA曲线进行排序，四种方案中肿瘤患者治疗后达到CR百分比最高的为伊立替康联合卡铂。敏感性分析及*meta*回归均没有改变结果。

#### 2.3.3 PR

我们对7项随机对照试验中的1, 187例患者及4种指南推荐的一线化疗方案的PR进行了网状*meta*分析。其中，伊立替康联合顺铂：共3项随机对照试验，309例患者；伊立替康联合卡铂：共2项随机对照试验，141例患者；依托泊苷联合顺铂：共5项随机对照试验，451例患者；依托泊苷联合卡铂：共4项随机对照试验，286例患者。结果表明，各方案PR之间没有明显的统计学差异。通过计算预测区间（95%PrI），四种方案结果仍无统计学差异。经异质性检验，各方案之间未见明显的异质性。根据SUCRA曲线进行排序，四种方案中肿瘤患者达到PR百分比最高的为伊立替康联合顺铂。敏感性分析及*meta*回归均没有改变结果。

#### 2.3.4 SD

我们对8项随机对照试验中的2, 169例患者及4种指南推荐的一线化疗方案的SD进行了网状*meta*分析。其中，伊立替康联合顺铂：共5项随机对照试验，854例患者；伊立替康联合卡铂：共2项随机对照试验，141例患者；依托泊苷联合顺铂：共7项随机对照试验，888例患者；依托泊苷联合卡铂：共4项随机对照试验，286例患者。结果表明，各方案SD之间没有明显的统计学差异。经计算95%PrI，四种方案结果仍无统计学差异。经异质性检验，各方案之间未见明显的异质性。根据SUCRA曲线进行排序，四种方案中肿瘤患者达到SD百分比最高的为伊立替康联合卡铂。敏感性分析及*meta*回归均没有改变结果。

#### 2.3.5 PD

我们对8项随机对照试验中的2, 169例患者及4种指南推荐的一线化疗方案的PD进行了网状*meta*分析。其中，伊立替康联合顺铂：共5项随机对照试验，854例患者；伊立替康联合卡铂：共2项随机对照试验，141例患者；依托泊苷联合顺铂：共7项随机对照试验，888例患者；依托泊苷联合卡铂：共4项随机对照试验，286例患者。结果表明，各方案PD之间没有明显的统计学差异。通过计算95%PrI，四种方案结果仍无统计学差异。经异质性检验，各方案之间未见明显的异质性。根据SUCRA曲线进行排序，四种方案治疗后肿瘤患者PD百分比最高的为依托泊苷联合顺铂，而伊立替康联合卡铂治疗后患者PD百分比最低。敏感性分析及*meta*回归均没有改变结果。

## 3 讨论

我们通过网状*meta*分析对目前指南所推荐的一线化疗方案的短期疗效进行了评估。一方面，我们通过整合间接证据（通过中间共同变量对非头对头实验进行比较），对尚缺乏直接证据（头对头试验）支持的化疗方案进行了间接比较，明确了其短期疗效。另一方面，通过比较不同方案之间ORR、CR、PR、SD及PD的差异，我们进一步明确了几种方案的有效性，并针对每个指标将不同的化疗方案的短期疗效进行了排序。我们的结果表明：①与依托泊苷联合卡铂相比，伊立替康联合卡铂使得肿瘤的CR明显提高，具有明显的统计学差异。②伊立替康联合卡铂治疗后CR明显优于依托泊苷联合顺铂，结果有明显的统计学差异。

既往有3篇传统的*meta*分析对依托泊苷和伊立替康分别联合铂类的方案进行了比较^[25-27]^。对于短期疗效，这几个研究仅对ORR进行了评估，但结论之间存在争议。争议的原因主要有：①双药方案中顺铂与卡铂之间的疗效差异；②样本量不足。我们的研究根据方案中铂类的不同进一步细分，并纳入了更多符合条件的研究，通过间接比较扩大样本量后，结果证明各方案间ORR没有明显的统计学差异，与最近的*meta*分析结果^[25]^一致。通过进一步对比各方案之间的CR、PR、SD及PD，我们发现除CR外，其余指标在各方案之间未见统计学差异。值得注意的是：①伊立替康联合卡铂在所有方案中，CR最佳，且分别与依托泊苷联合卡铂以及依托泊苷联合顺铂治疗后的CR比较，两组均有的统计学差异（前者结果经检验预测区间结果稳健，后者尚未稳健）。②其余指标在各个方案之间虽然未见明显的统计学差异，但经计算排序后发现，PD最低的方案为伊立替康联合卡铂；最高的为依托泊苷联合顺铂。③伊立替康联合卡铂方案与其他方案相比具有最高的ORR。以上三点研究证据表明，虽然部分方案ORR、PD以及CR的疗效排序是无明显统计学差异的，但是伊立替康联合卡铂因其治疗后最佳的CR、ORR及最低的PD，我们认为该方案可能具有较好的短期疗效。该方案远期疗效及其副作用还有待进一步评估。

该研究的不足在于：①伊立替康/依托泊苷联合卡铂方案的研究较少，较小的样本量可能影响到结果的准确性。②由于纳入研究较少，无法对发表偏倚进行评估。③由于研究间不能成环，故对研究间的不一致性无法做出系统评估。

综上所述，伊立替康联合卡铂方案治疗后达到CR的患者百分比高于依托泊苷联合卡铂以及依托泊苷联合顺铂。在所有指南推荐的一线方案中，伊立替康联合卡铂有最高的CR、ORR以及最低的PD；虽然对于短期疗效的排序不完全具有统计学差异，我们的结果倾向于支持伊立替康联合卡铂对于未经治疗的ED-SCLC具有最佳的短期疗效。
